# Re-Configuration of Sphingolipid Metabolism by Oncogenic Transformation

**DOI:** 10.3390/biom4010315

**Published:** 2014-03-14

**Authors:** Anthony S. Don, Xin Y. Lim, Timothy A. Couttas

**Affiliations:** Prince of Wales Clinical School, Faculty of Medicine, University of New South Wales, Sydney, NSW 2052, Australia; E-Mails: ameline_lxy@hotmail.com (X.Y.L.); t.couttas@unsw.edu.au (T.A.C.)

**Keywords:** sphingolipid, cancer, metabolism, sphingosine 1-phosphate, sphingosine kinase, ceramide, glucosylceramide, sphingomyelin, sphingomyelinase, glycolipid

## Abstract

The sphingolipids are one of the major lipid families in eukaryotes, incorporating a diverse array of structural variants that exert a powerful influence over cell fate and physiology. Increased expression of sphingosine kinase 1 (SPHK1), which catalyses the synthesis of the pro-survival, pro-angiogenic metabolite sphingosine 1-phosphate (S1P), is well established as a hallmark of multiple cancers. Metabolic alterations that reduce levels of the pro-apoptotic lipid ceramide, particularly its glucosylation by glucosylceramide synthase (GCS), have frequently been associated with cancer drug resistance. However, the simple notion that the balance between ceramide and S1P, often referred to as the sphingolipid rheostat, dictates cell survival contrasts with recent studies showing that highly potent and selective SPHK1 inhibitors do not affect cancer cell proliferation or survival, and studies demonstrating higher ceramide levels in some metastatic cancers. Recent reports have implicated other sphingolipid metabolic enzymes such as acid sphingomyelinase (ASM) more strongly in cancer pathogenesis, and highlight lysosomal sphingolipid metabolism as a possible weak point for therapeutic targeting in cancer. This review describes the evidence implicating different sphingolipid metabolic enzymes and their products in cancer pathogenesis, and suggests how newer systems-level approaches may improve our overall understanding of how oncogenic transformation reconfigures sphingolipid metabolism.

## 1. Introduction

Metabolic state changes, such as heightened glycolysis and lipid biosynthesis, are a hallmark of many, if not all, cancers [1,2]. Although the advent of personal genomics and large scale genome sequencing projects such as The Cancer Genome Atlas have yielded, and will continue to yield, important information on the many oncogenic mutations and chromosomal rearrangements that drive cancer initiation and evolution, it is becoming increasingly apparent that once the transformed state is acquired, cancer cells are capable of rapidly evolving to side-step these targeted therapeutics. One can argue that the return on investment for research into hallmark features that are common to many cancers will continue to yield greater improvements in treatment and survivorship than the current intense research into therapeutics that are targeted to specific driver mutations. This review will discuss current research concerning alterations to sphingolipid metabolism in cancer, and the significance of this for the cancer phenotype, in an attempt to gain a broad overview of established and emerging themes. Given the broad subject area, it is not possible to comprehensively review current pharmacology related to inhibition of specific sphingolipid metabolic enzymes in the context of cancer therapy. For comprehensive reviews on pharmacology related to inhibition of sphingosine kinases, ceramidases, sphingomyelinases, and glucosylceramide synthase, the reader is referred to recent reviews [3,4,5,6].

**Figure 1:**
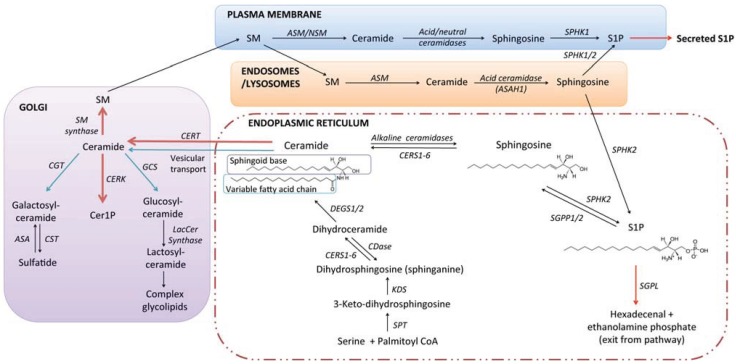
The sphingolipid pathway and basic structural units of sphingolipid biosynthesis. During *de novo* synthesis of sphingolipids in higher eukaryotes, the sphingoid base dihydrosphingosine (a.k.a. sphinganine) is formed by condensation of serine and palmitoyl-coenyzme A, catalysed by the serine palmitoyltransferase complex, followed by reduction of the resultant 3-keto-dihydrosphingosine. Transfer of a variable length fatty acid chain to the free amine group of dihydrosphingosine, a reaction catalysed by a family of six ceramide synthases (CERS1-6), forms dihydroceramide. Ceramides are subsequently formed by the desaturation of dihydroceramides, catalysed by dihydroceramide desaturases (DEGS1 and 2). Ceramides may then be transported to the Golgi, where functional headgroups are transferred to the primary hydroxyl, producing sphingomyelin, Cer1P, or glycolipids. Distinct sphingomyelinases and ceramidases catalyse the catabolism of SM and ceramide in distinct sub-cellular compartments. Catabolism of ceramides by ceramidases yields sphingosine, which can be recycled for new sphingolipid synthesis, or act as a substrate for phosphorylation by sphingosine kinases (SPHK1/2), yielding S1P. Irreversible cleavage of S1P by S1P lyase produces ethanolamine phosphate and hexadecenal, which can be recycled for new lipid biosynthesis. For more extensive reviews of sphingolipid metabolism and transport, the reader is referred to very comprehensive reviews [17,18].

The sphingolipids are one of the major lipid families in eukaryotes, distinguished from the more abundant phospholipids by the use of serine rather than glycerol as the headgroup to which the hydrophobic lipid tails are attached (Figure 1). Sphingolipids tend to associate more tightly with each other in cell membranes than phospholipids, thereby modulating the fluidity of membranes and forming the basis, together with cholesterol, for the densely packed regions of the membrane referred to as lipid rafts [7,8]. In addition to this fundamental membrane role, glycosphingolipids pattern the surface of the cell with a diverse array of oligosaccharide structures that dictate cell-cell interactions as well as modulating intracellular signalling. Other sphingolipids, such as ceramide, ceramide 1-phosphate (Cer1P), sphingosine, and sphingosine 1-phosphate (S1P), are relatively low abundance metabolites that act as primary or secondary signalling messengers and exert a powerful influence over cancer cell fate.

A unified picture describing how sphingolipid metabolism as a whole is reconfigured in cancer is yet to emerge, however it is now well established that many cancers are characterised by up-regulation of SPHK1, which catalyses the synthesis of the pro-survival, pro-angiogenic lipid signalling molecule S1P [4,9]. S1P is derived in two enzymatic steps from the central sphingolipid metabolite, ceramide, which is generally regarded as having tumour suppressive signalling properties [10,11]. The balance between these two metabolites has been termed the “sphingolipid rheostat” [12] and has attracted a great deal of attention in regard to its control over cancer cell survival. This hypothesis appears to be generally accurate on the basis of current evidence, but may need to be reconsidered in terms of how ceramide metabolism (including its conversion to S1P) fits into the broader context of lipid metabolism; and in light of recent data showing firstly that different ceramide variants appear to have very different roles in cell survival, autophagy, and metastasis, partly dependent on their cellular localisation [13]; and secondly that the most potent and selective SPHK1 inhibitors don’t affect cancer cell survival [14,15,16].

ASA: Arylsulfatase A; ASAH1: Acid ceramidase; ASM: Acid sphingomyelinase; Cer1P: Ceramide 1-phosphate; CERK: Ceramide kinase; CERS: Ceramide synthase; CERT: Ceramide transfer protein; CGT: Ceramide galactosyltransferase; CST: Cerebroside sulfotransferase; DEGS1/2: Dihydroceramide desaturase 1 or 2; GCS: Glucosylceramide synthase; KDS: 3-ketosphinganine reductase; LacCer synthase: Lactosylceramide synthase; NSM: Neutral sphingomyelinase; S1P: Sphingosine 1-phosphate; SGPL: S1P lyase; SGPP1/2: Sphingosine 1-phosphate phosphatase 1 or 2; SM: Sphingomyelin; SPHK1/2 : Sphingosine kinases 1 or 2; SPT: Serine palmitoyltransferase.

## 2. Sphingolipid Biosynthesis and Catabolism

The sphingolipid metabolic pathway in mammals begins with *de novo* biosynthesis of ceramide in the endoplasmic reticulum (ER) through a series of enzymatic reactions shown in Figure 1. Ceramide is the lipid “anchor” to which a range of different headgroup molecules may be attached. Transfer of a choline phosphate group produces the abundant plasma membrane lipid sphingomyelin; transfer of a phosphate yields the signalling molecule ceramide 1-phosphate; and transfer of glucose yields glucosylceramide, which can be further modified with sequential addition of different monosaccharide units to form the broad array of glycolipid structures encompassed within the ganglioside and globoside families [17,19].

Ceramide is not a single molecular structure; rather it is a group of molecules with a considerable degree of variation in the structure of the two lipid tails. The less variable lipid tail is referred to as the sphingoid base. In mammals the sphingoid base is most often 18 carbons in length, but 16 and 20 carbon variants exist [17]. In yeast the sphingoid base phytosphingosine, which carries an additional 4'-OH group, predominates. The variable length fatty acid that is transferred to dihydrosphingosine by ceramide synthases is usually from 14 to 26 carbons in length, with no or one double bond. The six different ceramide synthase isoforms preferentially transfer different fatty acids to dihydrosphingosine [17,20,21,22]. For example, CERS1 is selective for 18-carbon fatty acids (forming C18 ceramide), whilst CERS2 transfers very long chain fatty acid groups, ranging from 22 to 26 carbons in length. Thus, the ceramide composition of a given cell or tissue may vary according to the relative expression of the different CERS isoforms. Hydroxylation of the variable length fatty acid, commonly found in the brain and kidneys [23], further increases the complexity of possible ceramide structures and highlights the need for accurate and sensitive analytical approaches for sphingolipid quantification. Ceramide can also be formed through the “salvage” pathway, whereby S1P and sphingosine formed by the breakdown of more complex sphingolipids are recycled to ceramide, also utilising CERS enzymes.

In considering how sphingolipid metabolism is altered in cancer it is important to keep in mind its multi-compartmental nature (Figure 1). Sphingolipids formed at the plasma membrane are functionally distinct from those generated in the acidic organelles, mitochondria, endoplasmic reticulum, or nucleus [13]. Sphingolipid catabolism takes place primarily in the lysosomes, but catabolic enzymes such as sphingomyelinases and ceramidases may also be localised to other organelles (including the extracellular surface of the plasma membrane), where they generate lipids with distinctive signalling functions. Sphingolipids themselves regulate the formation and turnover of membrane compartments, examples being the regulation of autophagosome formation and turnover [24,25,26,27], regulation of exosome loading [28], and formation of the sphingolipid rich lipid raft domains that act as signalling platforms [7,29].

## 3. Signalling Roles of Ceramide, Sphingosine, and S1P

### 3.1. Ceramide

Ceramide levels increase in response to a diverse array of stimuli that induce cell death, including γ-irradiation [30], TNF/Fas receptor ligands [31,32,33], oxidative stress [33], and chemotherapeutics [29], and inhibiting ceramide formation in response to these stimuli retards the apoptotic process. Conversely, exogenous application of unnatural short chain ceramide analogues to cultured cells results in apoptosis [6,34]. The formation of ceramide in response to pro-apoptotic stimuli is often mediated through rapid hydrolysis of sphingomyelin (SM), resulting in the formation of ceramide-enriched membrane domains (ceramide “rafts”), which act as signaling platforms for pro-apoptotic mediators of the TNF superfamily [29,32,35]. These ceramide rich domains can also uncouple critically important signaling mediators such as the pro-survival kinase Akt/PKB from their membrane signalling domains [36]. Ceramides directly bind and stimulate the activity of the tumour suppressor protein phosphatase PP2A and the closely related phosphatase PP1 [37], as well as kinases PKCζ [38] and Kinase Suppressor of Ras (KSR) [39]. Direct binding of ceramide to the autophagosomal marker, Light Chain 3B (LC3B) directs autophagosomes to the mitochondrial membrane, promoting lethal mitophagy [26]. Long chain ceramides (C16 and C18) can also form channels in mitochondrial membranes, modulated by pro- and anti-apoptotic members of the Bcl-2 family, further emphasizing their pro-apoptotic signalling functions [40]. Contrary to the well accepted pro-apoptotic role for ceramide, its recruitment of KSR to the plasma membrane is necessary to stimulate the classical mitogen activated protein kinase/extracellular regulated kinase (ERK1/2) pathway in response to Epidermal Growth Factor (EGF) [41]. Studies demonstrating that exogenous ceramides induce cancer cell apoptosis gave rise to the hypothesis that direct delivery of ceramides could be used as a form of chemotherapy. Difficulties associated with the use of ceramides in living organisms include poor delivery (as ceramides are very hydrophobic) and the possibility that tumour cells are inherently better equipped to metabolise ceramide, discussed in the following section. Strategies to overcome the delivery problems include the use of nanoliposomal ceramide formulations [42,43,44] and short-chain ceramide analogues [6,10].

Although the focus in the literature has often been on pro-apoptotic functions of ceramide, knockouts of the different CERS enzymes have revealed critical roles for ceramides in diverse aspects of physiology that include maintenance of myelin and neuronal integrity [45,46], the skin permeability barrier [47], and liver physiology [48,49]. The ceramide-PKCζ interaction is also important in establishing cell polarity during neural stem cell differentiation [50,51,52], highlighting another tumour suppressive aspect of ceramide signalling.

### 3.2. Sphingosine and S1P

Ceramides are catabolised through the sequential action of ceramidases, which produce sphingosine; then sphingosine kinases (SPHK1 and 2), which phosphorylate sphingosine to produce S1P. Degradation of S1P by S1P lyase (SGPL) represents the only known “exit” from the sphingolipid pathway, allowing the 2-hexadecenal formed to be recycled into palmitoyl-coenzyme A [53,54]. It is possible that the sphingosine kinase/S1P arm of the pathway evolved for recycling of sphingolipids into fatty acids, however S1P is a potent signalling molecule that regulates a diverse range of physiological processes, ranging from control of neural stem cell maturation and neurotransmitter release in the brain, to control of lymphocyte entry into the blood stream, regulation of heart rate, and regulation of angiogenesis and endothelial barriers [55,56,57]. Sphingosine is itself a signalling lipid that directly binds to and regulates PKC isoforms, the 14-3-3 proteins [58], and acidic leucine-rich nuclear phosphoprotein-32A (ANP32A) [59]. These interactions promote tumour suppression and apoptosis [60,61].

**Figure 2:**
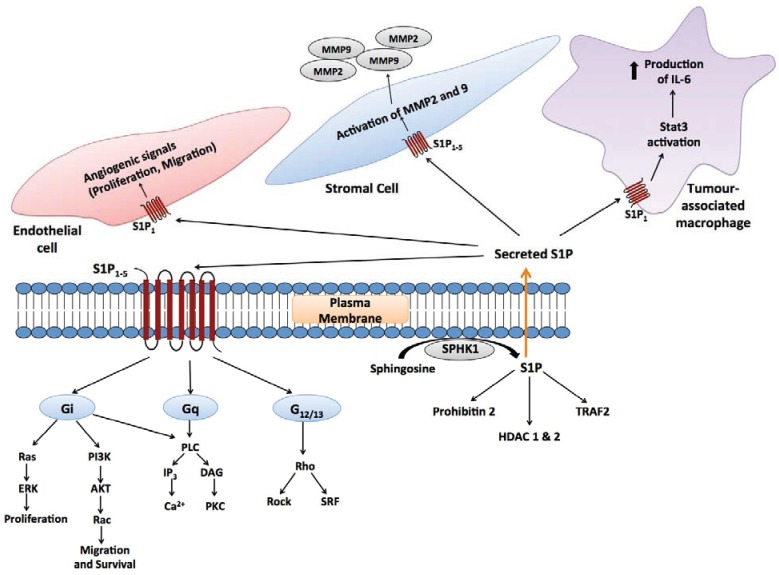
Autocrine and paracrine signalling mediated by S1P. Secreted S1P is capable of autocrine signalling, as well as signalling to other cells in the microenvironment (paracrine signalling). In the context of tumour biology, these cells include endothelial cells [68], stromal cells [69] and tumour-associated macrophages [70,71], as well as adaptive immune cells.

DAG: Diacylglycerol; ERK: extracellular regulated kinase; HDAC 1 & 2: Histone deacetylase 1 and 2; IL-6: Interleukin-6; IP_3_: Inositol triphosphate; MMP2 and 9: Matrix metalloproteinase 2 and 9; PI3K: phosphatidylinositol 3' kinase; PKC: Protein kinase C; Rock: Rho-associated protein kinase; S1P_1–5_: S1P receptors 1 to 5; SPHK1: Sphingosine kinase 1; SRF: Serum response factor; TRAF2: TNF receptor associated factor 2.

S1P signals with low nanomolar potency through its own family of five G-protein coupled receptors (GPCRs), S1P_1–5_ (Figure 2) [55,56,57]. Different S1P receptors couple to different G-proteins, thence transactivating multiple downstream signalling pathways, as reviewed in [4,56,57]. This signalling is essential for mammalian development, as mice that lack the ability to synthesize S1P (due to knockouts for both SPHK1 and 2), as well as mice that lack the S1P_1_ receptor die *in utero* with defects in vasculogenesis and neurogenesis [62]. Not only is formation of S1P through SPHK1 and 2 essential for development, but its degradation through SGPL is also essential, as mice lacking SGPL die approximately 1 month after birth with a variety of developmental defects that include severe dysregulation of lipid homeostasis in the liver [63] and systemic inflammation [64]. As seen with other sphingolipids, S1P binds to and directly modulates the activity of intracellular signalling proteins, including histone deacetylases 1 and 2 (HDAC1 and 2) in the nucleus [65], TNF receptor associated factor 2 (TRAF2) at the plasma membrane [66], and prohibitin-2 in mitochondria [67].

In direct contrast to ceramide and sphingosine, addition of S1P to cultured cells stimulates proliferation [72,73,74], and antagonises the effects of pro-apoptotic stimuli such as irradiation, chemotherapeutics, nutrient deprivation, and hypoxia [12,25,75,76]. S1P stimulates migration in a diverse range of cell types both *in vitro* and *in vivo*, mediated through S1P receptor signalling, most often demonstrated to be S1P_1_ and S1P_3_ [73,74,77,78,79]. Specific signalling through the S1P_2_ receptor antagonises cell migration in some settings [77,79,80] and promotes migration in others [81]. The physiological importance of S1P as a chemotactic factor has been well demonstrated in the case of lymphocyte circulation: S1P concentrations are in the high nanomolar range in plasma but are much lower in solid tissues, including lymph nodes. This allows S1P to act as an inducible signalling factor in solid tissues. High S1P levels in the blood and lymph are believed to act as a diffusible chemotactic signal, drawing lymphocytes from the lymph nodes into the circulation [82,83]. At the same time, tonic S1P_1_ stimulus of endothelial cells exposed to the circulation maintains endothelial barrier function [56,84].

## 4. Studies on Individual Enzymes and Sphingolipid Metabolites in Cancer

Studies on the role of individual sphingolipid metabolic enzymes in oncogenic transformation and cancer phenotype have focused primarily on SPHK1 and GCS, with a number of studies focusing on acid ceramidase and other enzymes. The literature on these enzymes in cancer is summarised below.

### 4.1. Sphingosine Kinase 1 (SPHK1)

SPHK1 is the most widely studied enzyme of sphingolipid metabolism in the context of cancer. There are four strong lines of evidence indicating that SPHK1 has an important role in cancer pathogenesis: (1) Increased SPHK1 expression has been demonstrated in a wide array of different cancers [4], and high level SPHK1 expression has been associated with increased cancer aggressiveness and poor survival outcomes in lung [85], breast [86,87], gastric [88], thyroid [89], prostate [90], and head and neck cancer cancer [91], and gliomas [92,93]; (2) Overexpression of SPHK1 transforms NIH3T3 fibroblasts, such that they are capable of forming tumours in mice [94,95]; (3) Loss of SPHK1 in mice inhibits genetically- or chemically-induced growth of colon and squamous cell carcinomas [96,97,98], as well as thymic lymphoma in p53 null mice [99], and promotes survival of mice with genetically-induced prostate cancer [100]. Similarly, knocking down SPHK1 with siRNA inhibits cancer cell proliferation and sensitizes cancer cells to radiotherapy or chemotherapeutics [101,102,103]; (4) A number of SPHK1 inhibitors and an antibody that specifically binds S1P inhibit tumour growth in mice [100,103,104,105,106,107].

There are a number of proposed mechanisms through which SPHK1 expression may be increased in cancers. Amplification of epidermal growth factor receptor (EGFR) signalling is a common event contributing to uncontrolled proliferation and survival in a wide range of cancers. EGF is known to stimulate SPHK1 expression and activity [108], as does expression of EGFRvIII, which is a truncated and constitutively active form of EGFR commonly found in glioblastomas [109]. SPHK1 expression is up-regulated by the BCR/Abl translocation that is characteristic of chronic myeloid leukaemia [110], and signalling through the S1P_2_ receptor stabilises the oncogenic BCR/Abl fusion protein [102]. Mutant B-Raf, the most common genetic lesion in melanoma, also up-regulates SPHK1 [69]. Oncogenic K-Ras activates SPHK1 post-translationally [111], whilst loss of p53 stabilises the protein, resulting in its up-regulation [99]. Mutant K-Ras stimulates SPHK1 activation through the Ras-Raf-MEK-ERK pathway and translocation of the enzyme to the plasma membrane, where it has ready access to its subtrate sphingosine [111]. Previous studies had demonstrated that phosphorylation of SPHK1 on S225 by ERK1/2 is necessary for its translocation to the plasma membrane [95], however the requirement for phosphorylation on S225 was not observed in the K-Ras study. Transfection with oncogenic H-Ras also increases SPHK1 activity [94]. Environmental influences that up-regulate SPHK1 expression and are important in the context of cancer include inflammatory cytokines, such as TNFα and IL-1β [112,113,114], and hypoxia [115,116]. Up-regulation of SPHK1 has also been demonstrated in multiple models of acquired drug resistance in cancer cells [117,118,119]. Increased SPHK1 expression in these models promotes cell survival via amplification of signalling through major nodes such as EGFR [117] and NFκB [119].

Increased SPHK1 expression supports cancer growth through direct stimulation of proliferation and survival pathways, and through modulation of the cancer microenvironment. In many respects, S1P generated by SPHK1 appears to act as a signal amplification factor downstream of growth factor and cytokine receptors, transactivating multiple pathways. SPHK1-S1P receptor signalling activates the ERK1/2 pathway, a well-established driver of cell proliferation and survival that is constitutively activated in many forms of cancer [12,73,81]. In one example, activation of ERK1/2 downstream of TGFβ signalling in esophageal cancer cells required SPHK1 signalling to G_i_ subunits via S1P_2_, which was shown to be necessary for migration and invasion in response to TGFβ [81]. In another example, SPHK1 activation downstream of the estrogen receptor generates plasma membrane S1P that activates the S1P_3_ receptor and thereby transactivates EGF receptor [120]. S1P signalling through S1P_4_ was shown to stimulate EGF receptor 2 (HER2) in breast cancer cells, which leads to ERK1/2 activation [121]. SPHK1 signalling also activates the PI3K-Akt pathway, another major cell survival and bioenergetic signalling pathway that is constitutively activated in cancer [98,103,122]. However, it should be noted that specific signalling through S1P_2_ can inhibit this pathway via activation of a Rho-ROCK-PTEN pathway [80,123]. The S1P_1_ receptor is a transcriptional target for signal transducer and activator of transcription 3 (Stat3), whose persistent activation is critical for the growth and survival of a range of cancers. In-turn, S1P_1_ signalling is necessary for persistent Stat3 activation, forming a positive feedback loop, both within the tumour cells and in the stromal support cells, that fuels the development and growth of melanoma or bladder tumour xenografts, as well as inflammation-induced colon cancer [71,124]. In an interesting contrast to these findings, expression of the S1P_1_ receptor suppresses glioblastoma growth and malignancy, despite the established association of SPHK1 with increased glioblastoma malignancy [92,125,126].

### 4.2. S1P Signalling in the Cancer Microenvironment

S1P is an essential angiogenic factor, required for *in vitro* angiogenesis even when other well established factors such as vascular endothelial growth factor (VEGF) are present [127,128,129,130]. As such, tumour angiogenesis in mice can be abrogated through systemic administration of an anti-S1P antibody [104], or through down-regulation of the S1P_1_ receptor, which is essential for the angiogenic properties of S1P [68,130]. S1P secretion by cancer cells also promotes lymphangiogenesis [127,131]. Cross-talk between S1P receptors, particularly S1P_1_, and the major angiogenic factors VEGF and angiopoietin-2 amplifies angiogenic and lymphangiogenic signalling [132,133]. Although S1P signalling promotes angiogenesis, specific signalling through S1P_2_ in endothelial cells and tumour-infiltrating myeloid cells has a counterbalancing effect, inhibiting tumour angiogenesis and tumour growth *in vivo* through inhibition of VEGF expression and MMP9 activity [123,134].

Not only has elevated SPHK1 in cancer tissues been implicated in disease progression, but systemic SPHK1 has been implicated in promoting metastasis through a feedback mechanism whereby systemic S1P, signalling through tumour cell S1P_2_ receptors, suppresses the expression of the protein breast carcinoma metastasis suppressor 1, which inhibits metastasis [100]. Thus, whilst S1P_2_ signalling has anti-tumourigenic properties in endothelial and myeloid cells [123], it has pro-tumourigenic properties in other contexts [100,102]. In another example of complex feedback between cancer cells and their microenvironment, SPHK1-S1P signalling from melanoma cells up-regulates SPHK1 expression in tumour associated fibroblasts. This stimulates their phenotypic transition to myofibroblasts, which was in-turn found to be an important factor driving melanoma growth in mice [69] (Figure 2).

Despite the vast body of evidence indicating an important role for SPHK1 in cancer cell proliferation and survival, two independent research teams from industry and academia recently described selective SPHK1 inhibitors—“1a” [15] and PF-543 [16]—which do not affect cancer cell proliferation or survival. Similarly, a dual SPHK1/2 inhibitor developed by Amgen, “Compound A”, did not inhibit cancer cell proliferation or survival and had no effect as a single agent in a mouse breast cancer xenograft model, at concentrations where S1P synthesis was effectively blocked [14]. The lack of effect of these recently developed inhibitors on cancer cell proliferation and survival sits in contrast to studies with earlier generation inhibitors, such as the dual SPHK1/2 inhibitors dimethylsphingosine [112,135,136] and SKI-II [105,106], and the SPHK1-selective inhibitor SK1-I [103,107]. In this regard, it should be noted that the newer inhibitors (1a, PF-543, and Compound A) are significantly more potent than their predecessors, with inhibitory constants in the nanomolar, rather than the micromolar range. Thus, the anti-tumour effects of SPHK1/2 inhibitors such as dimethylsphingosine (which is known to inhibit PKC [136]) and SKI-II may relate to off-target mechanisms [14]. Nonetheless, genetic studies definitely point to a significant requirement for SPHK1 in tumour growth [96,97,98,99,100]. In regard to the role of SPHK1 in tumour angiogenesis, compound 1a [15] potently inhibited glioblastoma-induced angiogenesis in an *in vitro* co-culture system [129], supporting studies with siRNA [130] and the less potent inhibitor SK1-I [127].

Only one publication has reported higher S1P levels in human cancer tissues [129]. S1P levels were significantly higher in both genetic and chemically-induced mouse models of colon adenocarcinoma, compared to the normal colonic mucosa [96,97]. However, S1P levels were reduced in a lipidomic study on metastatic pancreatic cancer, compared to normal tissue [137]. The lack of evidence for increased S1P in tumour samples is puzzling, but may relate to the specialized skills in lipid extraction and mass spectrometry needed for accurate quantification of S1P—a low abundance metabolite—in tissue samples. Further studies are needed to verify that high tumour SPHK1 expression translates into elevated tissue S1P levels. Significantly elevated plasma S1P levels have been demonstrated in breast [127], ovarian [138], and metastatic pancreatic [137] cancer patients. It remains to be determined whether this increase in circulating S1P is derived directly from tumour SPHK1 activity, or from host cells. The latter seems more likely, given the high abundance of S1P in the plasma (derived from circulating haematopoietic cells) compared to solid tissues [139,140]. This is supported by the observation that circulating S1P levels were reduced in prostate cancer patients when compared to control subjects or patients with benign hyperplasia [141], despite the reported up-regulation of SPHK1 in prostate cancer specimens [90].

### 4.3. Sphingosine Kinase 2 (SPHK2)

Reports on SPHK2 in the context of cancer are fewer and more conflicting. Early studies showed that overexpression of SPHK2 inhibits proliferation and promotes apoptosis, in direct contrast to SPHK1 overexpression [142,143]. However, silencing of SPHK2 has been reported to result in a more potent anti-proliferative response than silencing SPHK1 [92,144]. The SPHK2 inhibitor ABC294640, which also antagonises estrogen signalling [145], inhibits the growth of a number of different tumour cell lines *in vivo*, and has chemosensitizing properties [146,147,148,149,150]. Another recently described SPHK2 inhibitor shows similar anti-cancer properties [151], but the inhibitor described by Kharel *et al.* shows only very modest anti-proliferative activity [152]. Anti-proliferative properties of SPHK2 inhibitors may relate to an inhibition of autophagic turnover [24,148], loss of S1P receptor stimulus, or loss of signalling through intracellular S1P targets [65,67]. In this regard, downregulation of SPHK2 in breast and colon cancer cell lines reduced induction of the cell cycle inhibitor p21 and sensitized the cells to apoptosis induced with doxorubicin [153]. This is presumably mediated through the loss of SPHK2-S1P signalling to HDAC1/2 [65]. It remains to be demonstrated whether SPHK2 silencing blocks the proliferation of normal cell types, but it has been shown that loss of SPHK2 actually protects primary renal mesangial cells from pro-apoptotic stimuli through up-regulation of Bcl-X_L_ [154].

S1P produced by SPHK2 in breast tumour cells was reported to induce a pro-tumourigenic, anti-inflammatory phenotype (referred to as the M2 phenotype) in tumour-associated macrophages [70]. On the other hand, loss of SPHK2 in haematopoietic cells had a tumour-promoting effect in a colitis associated colon cancer model [71]. This was mediated through compensatory up-regulation of SPHK1, resulting in enhanced secretion of pro-inflammatory IL-6 by immune cells. These often conflicting studies indicate that the roles played by SPHK2 in cell proliferation and the tumour microenvironment are highly dependent on cell type and physiological context. However, the predominance of SPHK1 in the context of cancer is supported by the observation that SPHK1 is up-regulated, whilst SPHK2 is downregulated, as a function of glioma malignancy [129].

### 4.4. Sphingosine 1-Phosphate Lyase (SGPL)

Loss of SGPL expression has been described as a feature in colon and prostate cancers [155,156], and in melanoma cell lines compared to normal melanocytes [157]. However, higher SGPL expression was observed in fibroblasts following c-Src transformation [158], and in grade II and III gliomas compared to normal grey matter [129]. SGPL is a major sink for cellular S1P, so analogous to up-regulation of SPHK1, down-regulation of SGPL promotes higher S1P levels. Accordingly, SGPL expression enhances chemosensitivity and pro-apoptotic responses, whilst its downregulation blocks these phenotypes [155,156,157]. SGPL loss inhibits sphingolipid degradation, thereby increasing sphingosine and ceramide levels in mice [63]. Therefore, the cancer supportive effects of SGPL loss are presumably attributed to higher S1P rather than changes to ceramide levels.

### 4.5. Sphingosine 1-Phosphate Phosphatases

In the same study demonstrating loss of SGPL expression in colon carcinomas, loss of expression of both S1P phosphatases, SGPP1 and SGPP2 was demonstrated [155]. SGPP1 down-regulation has also been demonstrated in hepatocellular carcinomas [159], and a panel of melanoma cell lines, coinciding with up-regulated SPHK1 [69]. Our own study on malignant gliomas demonstrated that SGPP2, but not SGPP1, was strongly downregulated as a function of glioma malignancy, and that levels of SGPP2 were inversely correlated with S1P [129]. There is, therefore, some evidence that cancers are characterised not only by SPHK1 up-regulation, but also often by a down-regulation of the enzymes that degrade (*i.e.*, SGPL) or dephosphorylate S1P.

### 4.6. Ceramide and Ceramide Synthases

On the basis of the sphingolipid rheostat hypothesis, one would predict that levels of pro-apoptotic and pro-differentiative ceramide are reduced in human cancer tissues. In agreement with this, ceramide levels decrease in line with increasing glioma malignancy [129,160]. The loss of ceramide in gliomas was attributed entirely to loss of the C18 form of ceramide [129], mirroring earlier observations in head and neck squamous cell carcinoma (HNSCC) [161,162]. Reduced C18 ceramide in HNSCC was attributed to loss of CERS1 expression [161,162], but this was not the case for malignant gliomas [129]. C18 ceramide levels in HNSCC were inversely correlated with lymphovascular invasion and nodal metastasis, further emphasizing the tumour suppressive properties of this particular metabolite [161]. Subsequent *in vitro* experiments demonstrated a pro-apoptotic role for C18 ceramide, synthesized by the CERS1 enzyme, contrasting with a cytoprotective and pro-tumourigenic role for C16 ceramide synthesized by CERS6, in HNSCC cells [163,164]. In accordance with the differing effects of different ceramides on cancer phenotype, C16:0, C24:0, and C24:1 ceramides were significantly higher in HNSCC [161] and malignant breast cancer tissues [165]. Total ceramide levels were 12-fold higher in malignant compared to normal breast tissue, attributed to an up-regulation of CERS2, CERS4, and CERS6 [165], which was also observed in another study [166]. C16:0 ceramide also increased with increasing glioma malignancy [129], whilst both C16:0 and C24:1 ceramides were associated with increased nodal metastasis in pancreatic cancer [137]. Thus, although a pro-apoptotic role for C16 ceramide and CERS6 have been demonstrated in a number of publications using *in vitro* approaches [167,168,169], current evidence suggests that C16 ceramide is associated with an aggressive cancer phenotype, at-least in some cancer types, whilst C18 ceramide appears to impede malignancy. A specific pro-apoptotic role for C18 ceramide in promoting lethal mitophagy has recently been reported [26].

### 4.7. Acid Ceramidase

In the context of cancer, ceramidases occupy an interesting position as enzymes whose activity is firstly necessary to supply sufficient sphingosine as a substrate for SPHK1, and secondly acts as a means to reduce cellular ceramide levels. Acid ceramidase (ASAH1) up-regulation has been reported in prostate cancer, correlating with tumour grade [170,171,172], malignant gliomas [129], HNSCC [173], and T-cell large granular lymphocytic (LGL) leukaemia [174]. ASAH1 inhibition or downregulation leads to loss of cancer cell viability [173,174], anchorage-independent growth and metastatic potential [172], and sensitizes to cell death induced with Fas ligand [173] and radiotherapy [175]. Conversely, increased ASAH1 has been associated with resistance to Fas ligand [173] and radiotherapy [175]. These effects may be mediated through a reduction in ceramide content [172,173,175] and/or increasing availability of sphingosine for S1P synthesis [176,177]. These observations have given rise to efforts to design potent and specific ASAH1 inhibitors for use as chemo- and radiosensitizing agents [3,172].

ASAH1 expression has been positively correlated with Akt phosphorylation in prostate cancer tissues [176,177]. These authors identified a feedback mechanism whereby ASAH1 expression promotes Akt activation, and ASAH1-expressing cells are particularly sensitive to Akt inhibition [177]. Enhanced Akt activation through ASAH1 was attributed to increased downstream S1P synthesis, which releases the phosphatidylinositol 3' phosphatase PTEN from the nucleus [176,177]. PTEN is a tumour suppressor that inhibits Akt activation and is frequently mutated in many different cancer types. The ASAH1-SPHK1-S1P pathway also promotes prostate cancer cell invasiveness through up-regulation of the matrix protease Cathepsin B [178]. Even though ASAH1 and SPHK1 are sequential enzymes of the same pathway and have been functionally linked as described above, high ASAH1 expression has been associated with a positive outcome in epithelial ovarian and estrogen receptor positive breast cancers [179,180]. The opposite was suggested to be the case in prostate cancer, although numbers were not sufficient for robust statistical analysis [178].

### 4.8. Ceramide Kinase (CERK)

Phosphorylation of ceramide by CERK yields Cer1P, which is capable of stimulating macrophage proliferation and migration [181,182], and is well established as a co-factor for stimulation of cytosolic phospholipase A2 (cPLA2) [183,184]. Cer1P activation of cPLA2 is an important control point regulating the formation of inflammatory lipid metabolites such as prostaglandins. There is very little information on CERK in the context of cancer. PLA2 activity can promote or inhibit tumourigenesis, depending on the particular PLA2 isoform and the specific circumstances of the cancer [185]. CERK activity also acts as a sink for cellular ceramide, which would be predicted to have tumour-promoting effects. Accordingly, CERK has been shown to be necessary for proliferation and survival of A549 lung adenocarcinoma and SH-SY5Y neuroblastoma cells [186,187], and inhibition or silencing of CERK sensitizes SH-SY5Y cells to the cytotoxic properties of TNFα [188]. High CERK expression has been associated with a poor prognosis in breast cancer [189].

### 4.9. Sphingomyelin (SM) and Sphingomyelinases

SM is generally the most abundant sphingolipid in cells, and a major constituent of the plasma membrane [18]. A family of five sphingomyelinases hydrolyse SM to form ceramide. These enzymes are termed acid, neutral or alkaline sphingomyelinase, depending on the pH at which enzymatic activity is optimal. Neutral sphingomyelinases (NSM1 and 2; gene designations SMPD2 and SMPD3) are generally localised at the plasma membrane. Acid sphingomyelinase (ASM; gene designation SMPD1), is localised in acidic organelles, particularly the lysosomes, but can also be secreted, where it may hydrolyse SM on the extracellular surface of cell membranes [3,10]. Alkaline sphingomyelinase is localised to the liver and digestive tract, where it hydrolyses dietary sphingomyelin [190]. Pro-inflammatory and pro-apoptotic stimuli trigger the activity of acid and neutral sphingomyelinases. ASM activitation is the most studied sphingoymelinase isoform in this regard, shown to be an important component of the apoptotic programme initiated by TNFα, Fas, γ-irradiation, and chemotherapeutics [29,30,31,32,75,191]. In accord with these findings, decreased ASM expression has been published for a wide array of cancers, including colon, gastric, esophageal, renal, cervical, hepatocellular and lung cancers [158,159]. NSM activity has also been implicated in the execution of cell death in response to pro-apoptotic stimuli such as TNFα [33,192] and oxidative stress [33]. Loss of expression of NSM2 has been reported in colorectal, gastric, and lung cancers, and lympomas [158], and deletion or mutations in NSM2 that reduce enzyme activity have been reported in acute myeloid and lymphocytic leukaemias [192]. Striking and highly significant reductions in alkaline, neutral, and acidic sphingomyelinase activity have been demonstrated in colon carcinomas, as well as pre-malignant familial adenomatous polyposis lesions, compared to normal colonic tissue [193].

Mutation or changes in gene expression that result in loss of sphingomyelinase activity support the notion that cancer cells become resistant to cell death stimuli by reducing acute ceramide formation. Hence a recent study has proposed ASM administration as a potential therapeutic treatment for hepatocellular carcinomas [159]. Another recent report described an important role for host ASM in suppressing colon cancer and melonoma cell line metastases in the liver [194]. Mice lacking ASM failed to mobilise tumour inhibitory macrophages in the region of the tumour cells, and lacked tumour-associated hepatofibroblasts, which secrete the matrix metalloprotease inhibitor TIMP1. The authors suggested that the inhibitory effects of host ASM on tumour metastasis were mediated through S1P production, downstream of ASM, in the vicinity of the tumour cells. However, tumour suppression achieved through viral administration of ASM could not be reproduced by SPHK1 administration, raising the possibility that local ceramide formation enhances tumour cell rejection in the host liver. In contrast with these studies, a very recent study provided evidence that lower ASM activity in cancer cells creates a therapeutic window to induce lysosomal rupture using cationic amphilic drugs such as siramesine and desipramine (a clinical antidepressant), which inhibit lysosomal ASM [158]. ASM activity is required for lysosomal stability [195] and membrane fusion that facilitates vesicle and organelle turnover [196,197]. The authors suggest that the cytoprotective properties of ASM downregulation in cancer cells are related to the secreted form of the enyzme, which generates ceramide at the plasma membrane, whilst lysosomal ASM is needed for lysosomal integrity and cell viability.

In apparent contradiction to the reduced sphingomyelinase activity that has been reported in cancer, sphingomyelin levels have generally been reported as lower in tumours compared to normal tissues [198]. Transformation with oncogenic c-Src (Y527F) suppresses activity and expression of ASM, NSM2, and NSM associated factor; and increases expression of sphingoymelin synthase 2, whilst paradoxically reducing SM levels [158]. EGF receptor stimulation or overexpression, as well as K-Ras transformation, also downregulate ASM activity. Analogous to oncogenic c-Src, K-Ras transformation reduces cellular SM levels despite reduced ASM activity [158]. The basis for this paradox is unknown; however the therapeutic opportunity created by reduced SM levels in cancer was highlighted in a recent study showing that the anti-cancer agent 2-hydroxyoleic acid selectively kills cancer cells by stimulating SM synthases, which results in an elevation of SM levels [199]. In this study the increased SM content was proposed to alter plasma membrane signalling properties, favouring activation of the extrinsic cell death pathway and uncoupling oncogenic Ras signalling at the plasma membrane.

### 4.10. Glucosylceramide Synthase (GCS)

**Figure 3:**
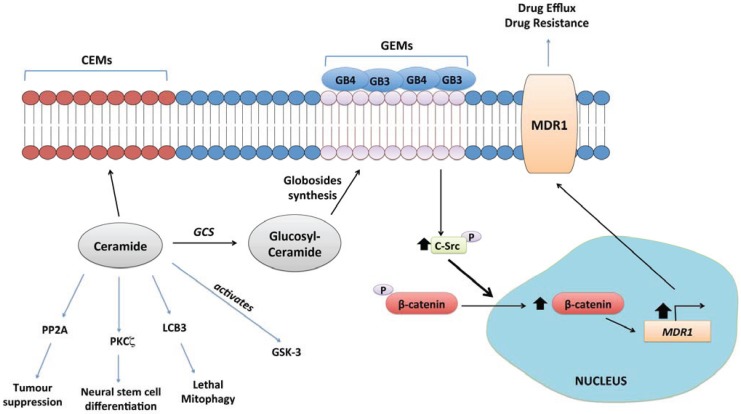
Glucosylceramide synthase (GCS) signalling. GCS is the gateway to synthesis of higher order sphingolipids, which form Glycolipid Enriched membrane Microdomains (GEM) on the extracellular leaflet of the plasma membrane [5]. Formation of GEMs enriched in globosides GB3 and GB4 has been linked to up-regulation of the multidrug resistance transporter, MDR1 (a.k.a. P-glycoprotein). This is mediated through activation of the tyrosine kinase c-Src by the GB3/GB4 domains, which promotes β-catenin translocation into the nucleus and transcriptional up-regulation of MDR1 [207,213]. GCS activity also reduces tumour suppressive ceramide levels.

CEM: Ceramide enriched microdomain; GB3 and 4: Globo-series glycosphingolipid 3 and 4; GCS: Glucosylceramide synthase; GEM: Glycosphingolipid enriched microdomain; GSK-3: Glycogen synthase kinase-3; LCB3: Light chain 3B; MDR1: Multidrug resistance protein 1; P: phosphate; PKCζ: Protein kinase Cζ; PP2A: Protein phosphatase 2A.

GCS (gene designation UGCG) catalyses the glucosylation of ceramide. Up-regulation of GCS has been well demonstrated as a mechanism through which cultured cancer cells acquire resistance to chemotherapeutics *in vitro* [5,200,201,202,203], and glucosylceramide accumulates in multi-drug resistant cells *in vitro* [203,204,205]. There is relatively little published evidence indicating that GCS inhibition or silencing blocks tumour growth *in vivo*, but a stabilised antisense oligonucleotide targeting GCS reduces the growth of adriamycin-resistant cancer cells and greatly sensitizes these cells to doxorubicin in a xenograft model [206,207]. At the simplest level, GCS activity decreases levels of pro-apoptotic ceramides [208,209]. Thus, inhibition or silencing of GCS was found to restore sensitivity in drug-resistant chronic myeloid leukaemia cells, both *in vitro* and *in vivo*, mediated through an increase in cellular ceramides that led to re-activation of the tumour suppressive kinase GSK-3 [209]. However, the further conversion of glucosylceramides to glycosphingolipids that promote anti-differentiative and pro-survival signalling is also a primary consideration with regard to the chemoresistance function of GCS (Figure 3). Resistance conferred by increased GCS expression has been linked in a number of studies to up-regulation of drug efflux transporters, particularly the ABCB1 transporter, also known as MDR1 [210,211,212]. Liu *et al.* have proposed a pathway whereby increased GCS expression results in increased expression of globo-series glycosphingolipids (GB3 and GB4) on the plasma membrane, which stabilises nuclear β-catenin via increased activation of c-Src. This pathway increases MDR1 expression through transcriptional up-regulation [207] and maintains the stemness of breast cancer stem cells [213].

Early studies with a small cohort of tumour specimens found that high glucosylceramide levels were associated with chemotherapy failure [204]. Both GCS and sphingoymelin synthase activities were very clearly and significantly increased in a small cohort of drug-resistant, as compared to sensitive, leukaemias of mixed type [208]. In a subsequent larger study, GCS expression was approximately 2-fold higher in drug-resistant *versus* drug-sensitive leukaemias, and was associated with higher MDR1 expression [212]. GCS expression is elevated in bladder cancers compared to normal bladder tissue, and high GCS expression was associated with a statistically significant reduction in survival time, possibly due to its higher expression in metastatic tumours [214]. GCS expression is also elevated in ER-positive breast tumours [215,216]. In one study, GCS expression was found to be higher in ER-positive breast cancers with lymph node metastases [216]. However, high GCS expression was very strongly associated with lower histological grade and lower proliferation in the other study [215].

### 4.11. Lactosylceramide and Complex Glycosphingolipids

As mentioned above, increased GCS activity may channel ceramides into biosynthesis of complex glycolipids that pattern the surface of the cell. Thus, globosides activate oncogenic Src kinase signalling, which is important for the maintenance of breast cancer stem cells [213]. Changes to the cell surface glycolipid signature in cancer, summarised in Table 1, are likely to be a feature of all cancers, but the specific changes vary with the individual grade, type, and molecular class of cancer. It is not possible to comprehensively review alterations to complex glycosphingolipids in cancer within this article, as that topic requires a separate review. The reader is referred to [217,218,219].

**Table 1:** Glycolipid Profiles in Cancer. Examples of altered glycosphingolipid expression in cancer are listed. The significance of these alterations for the cancer phenotype, if established, is also given.

Cancer type	Differential expression/functional significance of glycolipids	Reference
Breast Cancer	Elevated ganglioside GB5 and globohexaosylceramide (Globo H)	[220]
	in breast cancer cells with a stem cell phenotype	
Colon Cancer	Increased levels of lactosylceramide in association with up-regulation of human plasma membrane-associated sialidase (Neu3)	[221,222]
	Addition of lactosylceramide or transfection with Neu3 inhibits apoptosis, associated with increased Bcl-2 expression, in cultured colon cancer cells	
	Elevated expression of ganglioside GB3, which converts noninvasive epithelial cells into cells with an invasive and migratory phenotype	[223,224]
Glioblastoma Multiforme	Increased levels of simple ganglioside GM3, GD3;	[225,226]
	Decreased levels of complex gangliosides GT1b, GQ1b and GD1b	
	GD1b expression is inversely proportional to astrocytoma grade	
Glioma	Increased levels of ganglioside GD3 and lacto-series ganglioside 3'-isoLM1	[227,228]
Lung Cancer	Ganglioside GM2 important in maintaining growth of lung cancer cells in the presence of co-cultured fibroblasts	[229]
	Ganglioside GD2 elevated in small cell lung cancer	[230,231]
	Anti-GD2 antibody shown to suppress cell growth	
	and induced apoptosis in small cell lung cancer cells	
	Increased levels of ganglioside GD3 in small cell lung cancer	[232]
	Increased levels of Fucosyl-GM1 in small cell lung cancer	[232,233,234]
Medulloblastoma	Ganglioside GD1a, GM2 and GM3 shed into the	[235]
	microenvironment of Daoy medulloblastoma cell line	
Melanoma	Ganglioside GM2 elevated compared to normal melanocytes	[236]
	Increased levels of ganglioside GD2. Deposited in adhesion plaques, implicating GD2 as an adhesion mechanism in melanoma	[237, 238]
	Ganglioside GD3 is a predominant species found in melanoma,	[238,239]
	specifically deposited in adhesion plaques	
Neuroblastoma	Abundant expression of ganglioside GD2	[240,241]
Pancreatic Cancer	Elevated expression of ganglioside GB3 in pancreatic adenocarcinomas	[223, 224]
Renal Cell Carcinoma	Increased levels of lactosylceramide in granular cells	[242]
	and decreased levels in clear cells	
	Ganglioside GM3 elevated Wilms tumour	[242, 243]
	and in granular cells of renal cell carcinoma	
	Expression of ganglioside GD3 mediates apoptosis of	[244]
	activated T-cells in renal cell carcinoma	
Retinoblastoma	Increased levels of ganglioside GD2	[245]

The specificity of glycolipids displayed by particular cancers provides an attractive opportunity for immunotherapy [219]. Melanomas are characterised by high expression of the simple ganglioside GD3, which is not highly expressed in normal melanocytes or epithelium, making it a target of interest for immunotherapy [246,247]. Shedding of cell surface gangliosides such as GD3 into the tumour microenvironment may provide a mechanism through which cancer cells evade cytotoxic immune responses [248]. The example of GD3 is interesting, since it is generally considered a pro-apoptotic molecule, required for apoptosis triggered through the extrinsic Fas ligand pathway [249,250]. Thus, GD3 expression is a positive prognostic indicator in glioblastoma [251], and overexpressing GD3 synthase promotes apoptosis in glioblastoma cells [252]. This is despite the fact that glioblastoma is characterised by increased expression of the simple gangliosides GM3 and GD3, at the expense of more complex structures [225,227,251].

Complex glycosphingolipids are built upon glucosylceramide, but there is also a pathway for galactosylation of ceramide, which is particularly important for myelination of neurons [253,254]. Galactosylceramide sulfate, commonly referred to as sulfatide, is formed by reversible sulfation of galactosylceramide (Figure 1) and localises to the extracellular surface of the plasma membrane. Increased sulfatide levels in cancer *versus* non-cancerous control tissue have been demonstrated in various forms of ovarian cancer [255,256,257], and renal cell carcinoma [243]. Sulfatide levels increased with increasing grade and metastatic potential in a study of colorectal carcinomas [258]. The contribution made by increased sulfatide expression in these cancers is not established, but sulfatide is known to mediate cell adhesion [259].

## 5. Beyond the Single Enzyme: How is Sphingolipid Metabolism Reconfigured in Cancer?

The sphingolipid pathway is a good model system for whole pathway analysis, since it has a single biosynthetic entry point and a single catabolic “exit” point (Figure 1). Although the number of studies describing altered sphingolipid metabolites and/or their enzymes in cancer is large and growing, there is relatively little information regarding how sphingolipid metabolism at the broader level is re-configured by oncogenic transformation. Clearly, SPHK1 is up-regulated transcriptionally in a wide array of cancer types and this promotes carcinogenesis and/or tumour growth. This may be associated with other enzymatic changes that enhance ceramide catabolism to S1P and promote S1P signalling, such as enhanced ASAH1 [177,178], reduced S1P lyase or S1P phosphatase [129,155] expression, or enhanced S1P receptor expression. For example, microarray analysis of LGL leukaemia cells revealed concomitant up-regulation of ASM, ASAH1, SPHK1, and S1P_5_, which together would be expected to result in increased S1P synthesis and autocrine signalling [174].

In animals lacking p53, increased SPHK1 and S1P levels were associated with a decrease in ceramides [99]. Similarly, transformation with K-Ras increases S1P at the expense of ceramide [111]. However, gene expression profiling for other sphingolipid metabolic enzymes in the thymus of p53 deficient mice (thymus was studied due to the development of thymic lymphomas in these mice) demonstrated significant up-regulation of CERS1 and down-regulation of NSM mRNA, suggestive of oncogenic rewiring that favours overall sphingolipid synthesis. Post-translational up-regulation of SPHK1 presumably directs some of this increased sphingolipid synthesis towards S1P production. It was not determined whether loss of p53 altered the expression of GCS, but silencing GCS was shown to restore p53 functionality and sensitivity to chemotherapeutics in cells carrying oncogenic p53 mutations [260]. This indicates that GCS activity is critical for some of the oncogenic effects of p53 mutation, and the effects of GCS silencing appeared to be mediated through a restoration of cellular ceramide levels in these cells.

Viewed from the level of the pathway, observations on increased ceramide synthase expression [99,165,166] and increased levels of particular ceramides in metastatic cancers [137,161,165] could potentially be explained through a greater flux into lipid synthesis in general, as oncogenic transformation directs metabolism away from oxidative phosphorylation towards biosynthesis of lipids and other essential metabolites [2,261,262]. Loss of p53 is known to promote lipogenesis through activation of the pentose phosphate pathway [263] and the malic enzymes of the citric acid cycle [264]. Fatty acid synthase, the enzyme controlling fatty acid synthesis, is up-regulated in many forms of cancer [261], and in response to growth stimuli such as EGF [265]. Mass spectrometry measurements on multiple lipid classes have demonstrated that more aggressive cancer cells incorporate deuterated palmitate into lipid synthesis more rapidly than their less aggressive counterparts [266]. It would be interesting to fill in some of the gaps between entry into lipid synthesis and the increase in SPHK1 that is triggered by factors such as EGF [108] or loss of p53 [99], using comprehensive lipidomics coupled to gene expression profiling.

Increased synthesis of ceramides may also provide an avenue for the display of cell surface glycolipid signatures needed to maintain the cancer phenotype. In fact, increased ceramide synthesis in response to doxorubicin treatment was reported to transactivate the GCS gene promoter, indicating that glycolipid biosynthesis may be subject to feed-forward regulation in response to ceramide synthesis [267]. Mass spectrometry is theoretically capable of the simultaneous identification and quantification of basic sphingolipids and complex gangliosides and globosides in a single sample, allowing researchers to trace cancer cell sphingolipid metabolism from the synthesis of a particular form of ceramide through to SM, S1P, or a particular glycolipid structure. However, there are still relatively few mass spectrometry internal standards for the gangliosides and globosides. Rectifying this deficiency would help with the realisation of accurate wholistic models of sphingolipid metabolism.

Mathematical models of basic sphingolipid metabolism were first developed in the yeast *Saccharomyces cerevisiae* [268,269,270], and have more recently been described for macrophages stimulated with an endotoxin [271]. Important advantages with time-series pathway modelling approaches are their ability to compute particular reaction fluxes in isolation, and to identify modes of regulatory control that cannot be identified by measuring lipids at a given “snapshot” in time. Studies in yeast have demonstrated that the response to heat stress [270], or the switch from fermentative to oxidative metabolism [269], involves small but widespread and co-ordinated alterations to many sphingolipid metabolic enzymes, rather than large changes in a few key enzymes. Modelling how sphingolipid metabolism is reconfigured in response to oncogenic mutations can be achieved by following the metabolism of a metabolic tracer over time. Taking this approach, Mora *et al*. [272] followed the metabolism of fluorescently-labelled sphingomyelin over time, in the presence of inhibitors blocking different points of sphingolipid metabolism, and used this information to construct a mathematical model describing how sphingolipid metabolism is altered in malignant glioma cells compared to normal astrocytes. In agreement with the bulk of research to date, their model pointed to sphingosine kinase activity as good point for therapeutic intervention, as a key point of difference between glioma cells and normal astrocytes is the preferential channelling of sphingolipids into lysosomal S1P synthesis in the glioma cells. In normal astrocytes, sphingosine formed in the lysosomes is instead recycled into ceramide. These studies are in broad agreement with our own description of how sphingolipid metabolism and enzymes of the ceramide-S1P axis are reconfigured in human astrocytoma specimens [129]. The conclusion regarding lysosomal integrity as a weak point in cancer cells is well supported by studies on ASM, described above [158]. This study reveals the potential for mathematical modelling and flux studies to yield insights that take into account the compartmentalised nature of lipid metabolism.

With appropriate visualisation and processing tools, one may extrapolate from publicly available gene expression datasets to metabolite levels. Momin *et al.* described an approach specifically for the sphingolipid pathway that incorporates a simple method to visualise the pathway [273]. Publicly available gene expression data for sphingolipid metabolic enzymes was compared between invasive ductal breast carcinoma and normal ductal tissue. Their analysis predicted increased expression of complex gangliosides as well as particular globosides in the cancers, and the authors were able to confirm a number of these predictions from literature reports on glycosphingolipid levels (Table 1). Using the same approach, microarray data showed strong up-regulation of the genes regulating biosynthesis of sulfatide in ovarian cancer tissue samples, and this was confirmed at the level of the lipids using mass spectrometry [255]. Gene expression data can also be used to parametize reaction rates, facilitating the simulation of changes in metabolite levels in response to a particular perturbation, using mathematical modelling [269,274]. Application of this approach may yield testable hypotheses regarding how sphingolipid metabolic flux is altered in response to oncogenic mutations.

## 6. Conclusions

There is now a large body of literature describing changes to sphingolipid metabolism that characterise different cancers and are important for maintenance of the cancer phenotype. These studies have identified points for therapeutic targeting that induce cancer cell apoptosis, sensitize cancer cells to apoptotic stimuli, or block cancer cell support from the tumour microenvironment. The ability for high potency signalling molecules such as S1P to control host organ responses to the tumour cells appears to be strengthening the case for therapeutics targeting the SPHK1-S1P signalling system. The anti-S1P monoclonal antibody Asonep^TM^ is currently in Phase II clinical trials, whilst Phase I studies were recently completed for the SPHK1 and 2 inhibitor Safingol, which was one of the first described sphingosine kinase inhibitors [275]. Current evidence indicates that selection for high SPHK1 in cancer is driven both by oncogenic driver mutations and by environmental pressures such as hypoxia. Evidence for targeting of GCS is quite strong at the *in vitro* level and supported by studies with human tissue samples, but needs greater validation using pre-clinical models. Studies to date have tended to suggest that GCS up-regulation in cancer is driven by selection for drug resistance, but this may be a reflection of the hypothesis that has driven these studies. The recent revelation that inhibition of ASM with current lysosomal targeting drugs (cationic amphiphiles) shows selective toxicity towards cancer cells and inhibits tumour growth *in vivo* [158] is both interesting and promising from a therapeutic perspective, supported by the observation that increasing sphingomyelin synthase activity with 2-hydroxyoleic acid has similar anti-cancer efficacy [199].

Current research is increasingly directed towards gaining a better understanding of sphingolipid signalling in the context of sub-cellular localisation, and in terms of feedback between cancer cells and the host microenvironment. There appear to be a number of changes to sphingolipid metabolism that are common to multiple cancers, including the well-studied up-regulation of SPHK1, loss of ASM activity and altered lysosomal sphingolipid flux, and potentially an overall increase in sphingolipid biosynthesis, although the latter certainly requires further investigation. Changes to the cell surface glycolipid profile may be more specific for each molecular cancer sub-type and its tissue microenvironment. Future research looking at the co-ordinate regulation of multiple related enzymes and metabolites through flux modelling, transcriptomic, and lipidomic approaches will improve our overall understanding of metabolic reprogramming in transformed cells.
